# Enabling System Functionalities of Primary Care Practices for Team Dynamics in Transformation to Team-Based Care: A Qualitative Comparative Analysis (QCA)

**DOI:** 10.3390/healthcare11142018

**Published:** 2023-07-13

**Authors:** Lingrui Liu, Alyna T. Chien, Sara J. Singer

**Affiliations:** 1 Center for Evidence and Practice Improvement, Agency for Healthcare Research and Quality (AHRQ), U.S. Department of Health and Human Services, Rockville, MD 20857, USA; 2 Department of Pediatrics, Harvard Medical School, Boston, MA 02115, USA; alyna.chien@childrens.harvard.edu; 3 Division of General Pediatrics, Department of Pediatrics, Boston Children’s Hospital, Boston, MA 02115, USA; 4 Department of Medicine, Stanford University School of Medicine, Stanford, CA 94305, USA; sara.singer@stanford.edu; 5 Stanford Graduate School of Business, Stanford, CA 94305, USA

**Keywords:** primary care, transformation of team-based care, qualitative comparative analysis (QCA)

## Abstract

Team-based primary care has been shown to be an important initiative for transforming primary care to achieve whole-person care, enhance health equity, and reduce provider burnout. Organizational approaches have been explored to better implement team-based care but a thorough understanding of the role of system functions is lacking. We aimed to identify the combinations of system functionalities in primary care practices that most enable effective teamwork. We used a novel method, qualitative comparative analysis (QCA), to identify cross-case patterns in 19 primary care practices in the Harvard Academic Innovations Collaborative (AIC), an initiative for transforming primary care practices by establishing teams and implementing team-based care. QCA findings identified that primary care practices with strong team dynamics exhibited strengths in three operational care process functionalities, including management of abnormal test results, cancer screening and medication management for high-priority patients, care transitions, and in health information technology (HIT) functionality. HIT functionality alone was not sufficient to achieve the desired outcomes. System functionalities in a primary care practice that support physicians and their teams in identifying patients with urgent and complex acute illnesses requiring immediate response and care and overcoming barriers to collaboration within and across institutional settings, may be essential for sustaining strong team-based primary care.

## 1. Introduction

Well-implemented interprofessional team-based primary care has shown potential for improving the quality, efficiency, and effectiveness of healthcare delivery, patient outcomes, and provider work experience while reducing costs [1,2,3]. Implementing team-based primary care has been recognized internationally as a priority for reimagining primary care to improve both patient outcomes and the satisfaction of primary care providers. Team-based care may be particularly valuable in light of the increasing challenges of managing chronic diseases in rapidly aging populations, integrating behavioral health services into primary care, and dealing with the exacerbated burden and strains that primary care providers (PCPs) experience during public-health emergencies. In a team-based primary care delivery model, each team member’s unique expertise complements the others’, allowing each team member to make the most of their skills for better time management and workload distribution and to facilitate more comprehensive management of the care to meet patients’ health needs. Team-based care encourages optimizing the utilization of healthcare resources and promotes a patient-centered approach that improves the quality and efficiency of primary care services [1,4]. Primary care teams may appear to be highly stable, with team members working together for lengthy periods of time and dealing with a wide range of activities; however, in reality, implementing effective teams in primary care practices often involves overcoming various obstacles.

Organizational literature on effective teams suggests that, along with internal team factors (e.g., team configuration, clear roles, and directions), organizational-level factors, such as organizational structure, resources, system functionality, and contexts in which the team operates, are also important factors, shaping work team interactions and determining how well the team performs [5,6,7]. Studies have examined individual- and team-level interactions in healthcare teams and found collaboration, conflict resolution, engagement, and harmony to be most likely to impact staff-perceived team effectiveness [8,9]. In addition, literature on organizations and healthcare teams demonstrates that patterns of workgroup interactions and the effectiveness of teamwork outcomes are shaped not only by individual- or team-level interactions but also by organizational factors [7,10,11]. Work teams may find it difficult or impossible to obtain the organizational supports (e.g., human resource processes, system functionality, and technical support) required for effective teamwork [2,12,13]. In a rapidly changing, typically resource-limited ambulatory care setting, the transition to team-based primary care in practices may be challenging. Due to shift changes, patient transfers, and scheduling constraints, primary care practices experience frequent transitions among providers, which may lead to teamwork failures. In addition, for interprofessional teams, the professional hierarchy in medicine may impede open communication between individuals in high- and low-status roles [12]. When necessary, system functionalities are absent in primary care practices; this may create barriers that hinder individual- and team-level interactions in team processes. This may also negatively affect the development of psychosocial traits that are associated with team effectiveness, such as positive communication patterns; low levels of conflict; and high levels of collaboration, coordination, cooperation, and participation [8,11]. Without proper system functions in place to support providers in their clinical practice, such as decision support tools or access to critical patient information, providers may lack the means to deliver safe and high-quality care.

Although efforts have been made to explore organizational strategies for enhancing the implementation of team-based care, there remains a substantial knowledge gap concerning the roles of system functions—specifically, which combinations of system functions enable greater team effectiveness—acknowledging that, in practice, achieving optimal system functions in all aspects is challenging and may not be feasible. In this study, we captured and distinguished between two types of enabling system functionalities in primary care practices, operational care processes, and health information technology (HIT), and explored combinations of functionalities that could enable teams in primary care practices to perform efficiently and effectively.

We characterized operational care processes in primary care practices, such as appointment and referral management tracking for high-risk and high-priority patients, care transitions, and test-results management, as operational care process functionalities. We described automated reminders for cancer screening and other guideline-based interventions as HIT functionalities. These enabling functionalities create the conditions for constructive team dynamics to flourish. In theory, well-functioning operational care processes and HIT enable primary care practices to better anticipate, manage, track, share, document, and report items of information that are key contributors to positive team dynamics [8,14]. These enabling functionalities can enhance communication, information exchange, and conflict resolution among providers within a practice, building trust and fostering shared understanding and accountability, enabling appropriate allocation of responsibilities among personnel, and thereby encouraging providers to act and feel like a team [8,15].

However, despite the importance of implementing the team-based care model in primary care and the potential for organizational functionality to improve team effectiveness, there has been no rigorous investigation of which system processes or functionalities would enable well-implemented and high-functioning teams in primary care practices. In this study, we used an innovative analytic method, qualitative comparative analysis (QCA), to identify which system functionalities (i.e., operational care process and HIT functionalities) and which combinations of system functionalities could best support primary care practices through team-based care transformation and achieve better team dynamics as experienced by PCPs.

## 2. Methods

### 2.1. Context and Setting

This study was conducted in conjunction with the implementation evaluation efforts in the Harvard Academic Innovations Collaborative (AIC) and the “Comprehensive, Accessible, Reliable, Exceptional and Safe” (CARES) initiatives seeking to transform primary care practices by establishing teams and implementing team-based care [15,16,17,18]. Through the AIC, 19 academically affiliated medical practices in Massachusetts serve diverse patient populations committed to establishing care teams and delivering team-based care. They participated in triannual learning sessions, monthly webinars and data reporting, site visits, daily team huddles for information exchange about shared patients, and a series of qualitative and quantitative evaluation activities. Following two years of this foundational work, the CARES initiative sought to support high-functioning, AIC care teams to develop organizational capacities to become highly reliable primary care organizations. To facilitate the implementation of organization-level interventions, the research team evaluated practice-level system functionalities (i.e., operational care process and HIT functionalities) and the extent to which team-based care had been developed in AIC-CARES primary care practice sites.

### 2.2. Data Collection

#### 2.2.1. Practice Manager Survey on System Functionality

Between June and December 2014, a practice manager from each of the 19 participating primary care practices was surveyed to assess practice-level system functionalities, including operational care processes and HIT functionality. The operational care processes survey contained 40 items across eight domains to assess the extent to which the primary care practice operations included formal systems or processes for (1) appointment and referral management and tracking for high-risk/high-priority patients, (2) appointment and referral management and tracking for routine patients, (3) abnormal test-results management, (4) cancer screening and medication management for high-risk/high-priority patients, (5) cancer screening and medication management for routine patients, (6) patient-centered care, (7) patient safety, and (8) care transitions to emergency departments (EDs) or hospitals. Having a “formal system or process” meant having “an explicitly defined policy or standard operating procedure that is regularly followed by your practice’s staff or is embedded as part of the technology used in your office”. The HIT Functionality Survey included 42 items pertaining to HIT clinical decision support, HIT lab ordering, HIT imaging and procedure management, HIT meaningful use stage 2 requirements, HIT referrals, patient portals, recording aspects of patient complexity, and patient registries. Operational care process functionality survey items were rescaled from a four-point response scale *(1 = Not implemented, 2 = Considering, 3 = Partially implemented, 4 = Fully implemented)* onto a three-point scale with implementation = 0 for absent, 1 for partial, and 2 for full survey items regarding HIT functionality used a three-point response scale *(0 = Not implemented, 1 = Partially implemented, 2 = Fully implemented)*.

#### 2.2.2. Practice All-Staff Survey

In April 2015, 1716 patient-facing staff members (PCPs, other clinicians, and nonclinicians) across the 19 participating primary care practice sites that had established team-based care in the Harvard AIC–CARES initiatives were surveyed via work emails. The survey included psychometrically verified items assessing providers’ experience of team dynamics (the outcome of interest in this study).

The team dynamics in primary care practices reflect the level of team-based care. We used the Team Dynamics in Primary Care Survey, composed of 33 items pertaining to eight factors, to comprehensively assess the extent to which healthcare professionals perceive and consider themselves to be part of a “team” when providing patient care in ambulatory settings. This instrument is based on a previously validated primary care team-dynamics model with seven factors (conditions for team effectiveness, shared understanding, processes for accountability, processes for communication and information exchange, processes for conflict resolution, acting and feeling like a team, and perceived team effectiveness) [15]. The original version was modified to include one additional factor related to learning processes. Research suggests that these eight team dynamics facilitate the work of teams [17]. These survey items used a 5-point Likert scale (5 = *strongly agree, 4 = agree, 3 = neither agree nor disagree, 2 = disagree, and 1 = strongly disagree*). (See Appendix A for survey details).

The study sample included PCPs working in the 19 AIC–CARES primary care practice sites. In this study, we included as PCPs all individuals serving in clinical role categories defined in the Harvard AIC initiative, including attending physicians (physicians, nurse practitioners, and physician assistants), resident physicians, and other patient-facing clinical personnel (including specialist physicians, mental and behavioral health providers, medical assistants, registered and other nonregistered nurses, and allied health professionals). Respondents were asked in the survey’s opening question to define their primary care team by selecting the types of people with whom they frequently collaborate when providing patient care at their practice. They were then asked to consider this group of people as their “team” in answering subsequent questions.

### 2.3. Statistical Analysis

#### 2.3.1. Descriptive Analysis

We first verified survey properties (including nonresponse, means, and variance) and performed standard descriptive analysis to assess the demographic characteristics of the survey sample (age, gender, race/ethnicity, and type of job/profession) and characterize practice sites by factors such as practice-site size, operational care processes and HIT functionality, and team dynamics. See Table 1 and Table 2 for descriptive statistics.

From each of the 19 practice sites, one practice manager completed the survey questionnaire (100% response rate), which assessed the practice-level operational care process and HIT functionality. The proportion of missing data for team dynamics in the all-staff survey was minimal (less than 1% of the data). There was no instance in which an individual failed to respond to more than half of the items for each factor. We calculated an overall team-dynamics score for each individual respondent by averaging the items that each respondent answered across 33 items reflecting the eight team-dynamics domains. We then combined responses from the all-staff and practice-manager surveys by first collapsing the individual responses to the mean for the respective sites and then merging the site-level values for the all-staff survey with those for the practice-manager survey. The final analytic file (shown in Table 2) contained 19 rows of observations (each row representing a primary care practice site).

#### 2.3.2. Qualitative Comparative Analysis

To identify cross-case patterns in primary care practices that would indicate which combinations of system functionalities would most enable practices to establish and implement team-based care, we employed QCA to summarize and identify patterns in the data [6,19,20,21,22]. Unlike conventional statistical approaches, which examine the “net effects” of single variables on the outcome of interest, QCA methodology is based on Boolean logic that compares cases to identify necessary and sufficient conditions (“variables” in conventional statistics) for an outcome and identifies specific combinations of these conditions that may be significant and most conducive to the occurrence of the outcome [6,19,20,21,22]. Originating from political science and sociology research, the QCA method has been utilized in various fields of social science, including business, management, and environmental studies. Although there has been a recent increase in the application of QCA in evaluating public health interventions [23,24,25,26,27], the method is still relatively new in healthcare management and organizational studies. QCA is well-suited to this project and has been shown to have utility in addressing questions in this field, [18,24,25,26,27] which typically have small to medium sample sizes (10–30 cases, e.g., physician practices and hospitals); ask questions at the organizational, system, or practice level; and frequently emphasize practical implications for healthcare managers and stakeholders.

To conduct QCA, we followed an analysis process outlined in prior literature [6,19,20,21,22]. The technical details are described by the authors in an earlier study [18] and are available from the authors upon request. Overall, we performed five steps. A visualized figure presenting these steps is available in Appendix B. First, we used the direct method of calibration to transform the raw scores into a rescaled format ranging from 0 to 1, measuring the extent to which this condition was met for each practice site, which was the unit of analysis [21]. See Table 3 for the calibration scoring. A score of 0 indicates the condition was not fully met, while a score of 1 indicates the condition was fully met. The calibration thresholds are determined by the statistical or substantive properties of each variable. We recalibrated and computed the final calibrated score for each variable in consultation with QCA methodology specialists. See Table 4 for the calibrated data for the analysis. Second, we used the calibrated set membership scores to first identify the necessary conditions for “greater team dynamics” in the practice sites and then to examine the sufficient conditions. For each combination of conditions (“configurations” in QCA terminology), we started by looking at the bivariate relationship that tests each single condition with the outcome of interest. We added conditions one by one, creating combinations of conditions, to examine the specific combinations that could be conducive to the outcomes while meeting the goodness-of-fit criteria (i.e., a consistency score higher than 0.8, indicating strong association between the conditions and outcomes, and the highest coverage score, indicating that the largest number of sites have this specific bundle) [21,22,25,28]. Additionally, we conducted a number of robustness checks to evaluate the robustness of the findings when employing alternative calibration threshold specifications [20]. The tests for robustness revealed minimal modifications to a specific number of cases, while the fundamental conditions and main bundle did not change. Consequently, the interpretation and conclusions remained unchanged.

The first author, who had completed training in the QCA methodology, followed a validated process reported in the literature to perform the analyses [19,22]. The first author discussed the results with the coauthors, who have strengths in organizational theory and survey design and clinical expertise in primary care, and consulted clinicians and practice managers from the participating practices. One QCA methodologist performed reviews of the analytical approach. This study used software including Stata/MP 15.0 and fuzzy-set QCA 3.0.

## 3. Results

### 3.1. Characteristics of Participating Primary Care Providers

Overall, the analytical sample in this study included 854 PCPs (79%) out of 1082 respondents among the 1716 surveyed (response rate 63) from the 19 participating AIC–CARES practice sites. This number included 238 attending physicians, 249 resident physicians, and 367 other patient-facing clinical professionals.

### 3.2. Characteristics of Participating Primary Care Practices

Table 1 characterizes the practices that established team-based primary care models in the Academic Innovations Collaborative. Of the 19, 11 were community-based practice sites and 8 were hospital-based sites. Overall, female PCPs (72%) outnumbered male PCPs, with the majority being between the ages of 36 and 50. Furthermore, Hispanic and African American providers accounted for only around 13% of all providers. A large number of respondents practiced in hospital-based locations (520 PCPs) compared to community-based locations (334 PCPs).

Table 2 shows the assessment of practice-level operational care process and HIT functionalities, as well as team dynamics experienced by PCPs at each site. Each row of observations corresponds to a single primary care site. On a three-point scale (0 = absent, 1 = partially adopted, 2 = fully implemented), practices overall had a higher score on appointment and referral management and tracking for high-risk/high-priority patients over routine patients (1.26 vs. 0.91, respectively). Cancer screening and medication management received poorer scores for functionality for high-risk/high-priority patients compared to routine patients (1.18 vs. 1.44, respectively). The processes for managing abnormal test results received the highest rating (1.63), followed by processes for ensuring patient safety (1.47), supporting care transitions (1.39), and promoting patient-centered care (1.35). HIT features were rated by respondents as partially implemented, with an average score of 1.36 on a three-point scale (0 = not implemented, 1 = partially implemented, 2 = fully implemented). Respondents rated their team dynamics experiences on average as 3.68 (SD 0.63) on a scale of 1 to 5, indicating slightly above neutral response (*1 = Strongly Disagree/Very Dissatisfied/Never to 5 = Strongly Agree/Very Satisfied/Always*).

### 3.3. Combinations of Enabling Conditions That Promote Team Dynamics

Table 3 presents the enabling conditions (factors) of system functionalities that were characterized in this study, including operational care process functionality and HIT functionality and the calibration scale that was used to investigate key combinations of conditions (“recipes”) that could be conducive to greater team dynamics among PCPs in the transformation of team-based primary care. The calibrated scores are shown in Table 4 for all the conditions and outcomes for each primary care practice, indicating the extent to which this condition is present for each practice, with *1 = condition fully met* and *0 = condition fully unmet*.

Table 5 shows the results from QCA that summarize and identify the patterns across the 19 primary care practice sites. We identified the combinations (“recipes”) of enabling conditions (system functionalities) that, when present in the primary care practice, almost always correlated with the desired outcome (great team dynamics among PCPs that respondents view as part of their team).

In the transformation of team-based primary care practices, two recipes were associated with excellent team relationships. Each is composed of three formal processes, including (1) managing abnormal test results, (2) managing cancer screening (e.g., priority colonoscopies and mammograms) and medication for high-risk/high-priority patients, and (3) ensuring care transitions to EDs or hospitals, combined with the HIT functionalities of managing automated reminders for cancer screening or other guideline-based interventions. These *core* enabling functions, in conjunction with *either* a formal process supporting teams providing patient-centered care (recipe 1) *or* a formal process promoting patient safety (recipe 2), may best support practices in achieving better team dynamics among PCPs. The QCA methodology uses two measures of goodness-of-fit: consistency, on a scale of 0–1, measures the strength of the association, with a threshold of 0.8 being generally acceptable, and coverage, on a scale of 0–1, indicates the fraction of cases in the empirical data to which the relationship applies, with higher coverage indicating a greater number of empirical cases in the data to which the relationship applies. Recipe 1 has consistency and coverage values of 0.94 and 0.37, whereas recipe 2 has consistency and coverage values of 0.97 and 0.38. The total solution has consistency and coverage values of 0.95 and 0.48. High consistency and moderate coverage indicate that, compared to primary care practices that do not meet either of these conditions, scenarios in which one of these two combinations of enabling system functionalities is present would be most conducive to stronger team dynamics in team-based primary care transformation.

## 4. Discussion

We used a novel method to identify patterns that promoted teamwork and team-based primary care delivery among 19 practice sites participating in the Harvard AIC–CARES initiatives. Applying QCA, we examined the cross-case patterns and investigated practical “recipes” (combinations of system functionalities that present the greatest potential for enhancing PCPs’ team dynamics) to help healthcare managers and stakeholders prioritize resources and investment. The findings indicated that primary care practices with high-functioning teams exhibited strengths in three operational care process functionalities: managing abnormal test results, managing cancer screening and medications for high-risk/high-priority patients, and supporting care transitions, along with HIT functionality. These findings suggest that the performance of physicians and their care teams and teamwork outcomes are greatly influenced by how well they address the challenges encountered when dealing with patients who present with urgent and complex acute illnesses that require immediate actions or care. Performance and teamwork outcomes are also influenced by how well providers overcome the glitches and obstacles that frequently arise during collaborations within and across institutional settings. Our findings also suggest that key system activities that can enable teams to meet their most crucial needs and provide teams with means to achieve their objectives in delivering care are critical to establishing successful teams and sustaining strong team-based primary care.

Our results suggest that select operational care processes are key. In particular, they suggest that high-functioning primary care requires systems that support primary care physicians and their care teams in identifying urgent or complex acute illnesses, addressing high-risk/high-priority patients with complex comorbidities or special demands, and managing collaborations among primary care personnel across institutional settings. These in turn are essential for reducing the provider burden and facilitating team-based care, which is often unreliable in care delivery [29]. For example, when the primary care practice has a formal process in place for managing abnormal test results, the process ensures that a clinician reviews the results, notifies the patient, discusses the results with the patient, and follows-up as needed. When a practice site has a process for managing cancer screening tests (e.g., mammograms or colonoscopies) for high-priority patients, it guarantees that tests are completed, results are followed-up, and medications are adjusted as necessary. The care transition process ensures that patient information is transmitted to and received from admitting hospitals, that patients are followed during their hospitalizations, and that discharge plans and follow-up appointments are coordinated. Treating high-risk patients and addressing care transitions often requires interprofessional collaboration across institutional settings (e.g., EDs, specialist clinics, and hospitals), in which communication and coordination challenges abound.

Formalizing such processes reduces wasted time and duplication of routine tasks and offloads nonspecialized tasks from PCPs to other personnel within the practice, allowing PCPs to focus on tasks at which they are uniquely skilled (e.g., decision-making with patients), using their time more efficiently, and letting other clinical team members (e.g., physician assistants and nurse practitioners) perform other tasks (e.g., conduct routine physical exams, order and interpret the more mundane tests, counsel on preventive healthcare, and write prescriptions). Other clinical team members can assist in procuring information that physicians do not have time to obtain but that could be essential for diagnosis and treatment decisions [14]. Nonphysician clinical team members may, in turn, have better training and specific skills for duties such as social work, nutrition, and pharmacy.

Recommendations for improving operational care process functionalities include promoting shared decision-making, the collection of detailed clinical and socioeconomic information about patients, patient engagement, and participatory decision-making [30,31]. Encouraging patient engagement involves actively involving patients in discussions about their treatment options and goals. It is crucial for achieving better patient-centered care and improved patient satisfaction to involve patients in decision-making processes and ensure they are well informed about their options and potential risks and benefits. Gathering clinical and socioeconomic information about patients could provide healthcare teams with information to tailor treatment plans to meet the needs of patients. Studies also recommend that primary care practice sites ensure accessible patient information with prioritized and synthesized main messages, offer peer support and training for PCPs on how to engage patients in difficult discussions, and augment the usability of HIT and computerized decision tools, such as user-friendly interfaces, clear prompts, and decision support features, to aid PCPs in navigating complex screening recommendations [30,32].

Our findings show that HIT does not work in isolation and that its effect on the immediate outcome of team dynamics is not sufficient. Rather, it must act together with the other identified conditions to produce positive effects for improving provider experiences. This finding emphasizes the human-centric nature of patient care and underscores the significance of considering the holistic interaction between HIT and other sufficient conditions to achieve desirable outcomes in healthcare work teams. This insight may enable healthcare-system managers, executive leaders, and other stakeholders to make better decisions about where to invest the resources most likely to enhance the experience of frontline healthcare workers in their work. The ability to direct resources in ways that will achieve the greatest benefit is crucial, especially during times of public-health emergencies due to the limited resources, rapid evolution, and complexity of the challenges providers face.

A primary care practice with better formal care processes and HIT system functionality is better able to anticipate, manage, track, share, document, and report information. These functionalities can enhance communication, information exchange, and conflict resolution among team members, which can help team members build trust and establish shared understanding and accountability, further encouraging individual members within the practice to feel and act like a team. When these enabling functions work well, the team can perform more efficiently and effectively and deliver care with improved outcomes for both patients and providers.

We have reported other research applications of QCA in the Harvard AIC–CARES program evaluation, [18,33,34] which examined the organizational capabilities of primary care practices for improved practice transformation outcomes and improved the work life of providers and staff. This earlier research found that establishing high-functioning teams and implementing team-based care, together with creating and sustaining a strong safety culture, acted as the core conditions for enhancing PCPs’ clinical work satisfaction. Following the earlier research findings, the present study sought to reveal which system functionalities and combinations of functionalities primary care practices could improve to best support their transformation to team-based care. To our knowledge, this is the first paper that explores the combinations of conditions (system-level functionalities of primary care practices) that are conducive to this outcome (strong team dynamics among the group that PCPs view as their team). Prior research has included some of these variables, employing conventional statistical methods based on linear algebra and statistical inference to investigate measures for increasing teamwork in primary care [5,13]. Conventional regression and statistical analyses provide a perspective on the “net effects” of a single variable on the outcome but are limited in their ability to provide practical information for answering “configurational” questions (i.e., how different elements or combinations of a system interact and combine to produce specific outcomes or patterns of interest), which are often encountered in research on implementing interventions in healthcare. Our research demonstrates an alternate method for investigating cross-case patterns, which could enhance team-based care in ambulatory primary care settings. This work provides a new perspective for stakeholders with a practical view and contributes insight into the complex interactions within healthcare teams in primary care settings.

Some limitations of our research need to be considered. First, as we noted in a previous work, the QCA approach may be prone to type I errors, as claimed by some statistical methodologists [18,35]. Second, the surveys were cross sectional, which precluded examination of causal links. In this work, we do not attempt to draw causal inferences; rather, we adopt an alternative implementation-oriented methodology. Our findings should be confirmed quantitatively on a larger scale in future studies. In addition, we investigate the combinations of system functions that are favorable to the occurrence of strong team dynamics but we do not identify the barriers to enabling system functionalities for implementing team-based care; these barriers need to be considered in future research. Furthermore, we acknowledge that this work uses data from surveys administered in 2015, and some of the data may have changed over time. In practice, and especially in light of the COVID-19 pandemic, PCPs have experienced extraordinary burdens and stresses. Still, the practical implications of this study could apply beyond the context of the AIC initiative, assisting healthcare managers and stakeholders with decision-making and providing an empirical point of view to enable better prioritization of investment and resources for implementing team-based primary care.

Future studies may focus on refining and expanding the utility of the methods applied in this study in healthcare organizations and management studies. Researchers could employ a mixed-method approach to design qualitative and quantitative data collection suitable for the QCA method. To translate findings into practice and to promote evidence-based practices, future research will need to develop practical guidelines, protocols, and intervention strategies that primary care practices can adopt to ensure the functionalities of key operational care processes and HIT for improved team effectiveness and better team outcomes.

## 5. Conclusions

Recognizing that it is not always feasible to improve practices for optimal system functionalities at all levels and in all parts simultaneously, we approached this study from a practical perspective geared towards informing healthcare administrators and related stakeholders with regard to decision-making and resource allocation. The literature suggests that combinations of practice-level organizational factors, rather than isolated individual factors, seem to be important for practice transformation to team-based care. Our research confirms and further explores the combinations of system functionalities that practice administrators might emphasize to achieve favorable team dynamics. Given that changes in established practices, such as formal care processes or HIT functionality, are often difficult to implement, primary care practices would benefit from knowing which core processes or functionalities, when combined, have the greatest potential for positive impact. Practice managers, policymakers, and other stakeholders can concentrate on these areas to boost the team-based primary care model, enhance care delivery, and encourage PCP retention.

## Figures and Tables

**Table 1:** Characteristics of primary care providers and practices engaged in establishing team-based care via the Academic Innovations Collaborative ^a^.

	Total (n = 19)	Community-Based (n = 11)	Hospital-Based (n = 8)
**Characteristics of Primary Care Providers (PCPs)** ^b^	N = 854	N = 334	N = 520
Gender—female, n (%)	613 (72%)	263 (79%)	350 (67%)
Race ^c^, n (%)			
White	379 (44%)	148 (44%)	231 (44%)
Hispanic	76 (9%)	50 (15%)	26 (5%)
African American	37 (4%)	21 (6%)	16 (3%)
Age ^c^, n (%)			
Under 30	61 (7%)	35 (10%)	26 (5%)
30–35	72 (8%)	41 (12%)	31 (6%)
36–50	192 (22%)	84 (25%)	108 (21%)
Above 50	194 (23%)	78 (23%)	116 (22%)
Declined to answer	335 (39%)	96 (29%)	239 (46%)
**Characteristics of Primary Care Practice Sites**			
Practice size			
MDs ^d^, mean number (range)	11 (2–36)	8 (4–11)	17 (2–36)
Attending physicians, number of total surveyed (%)	238 (28%)	96 (29%)	142 (27%)
Resident physicians, number of total surveyed (%)	249 (29%)	50 (15%)	199 (38%)
Other patient-facing providers, number of total surveyed (e.g., nurses, front-desk clerks) (%)	367 (43%)	188 (56%)	179 (34%)
**Operational Care Process Functionality ^e^, mean (sd) with implementation = 0 for absent, 1 for partial, or 2 for full**			
(1) Appointment and referral management and tracking for high-risk/high-priority patients	1.26 (0.54)	1.27 (0.55)	1.25 (0.55)
(2) Appointment and referral management and tracking for routine patients	0.91 (0.44)	0.97 (0.42)	0.83 (0.50)
(3) Abnormal test-results management	1.63 (0.39)	1.68 (0.32)	1.56 (0.50)
(4) Cancer screening and medication management for high-risk/high-priority patients	1.18 (0.63)	1.30 (0.55)	1.03 (0.75)
(5) Cancer screening and medication management for routine patients	1.44 (0.46)	1.40 (0.46)	1.5 (0.5)
(6) Patient-centered care	1.35 (0.42)	1.47 (0.34)	1.18 (0.47)
(7) Patient safety	1.47 (0.32)	1.42 (0.37)	1.54 (0.25)
(8) Care transitions (to EDs ^†^ or hospitals)	1.39 (0.33)	1.48 (0.34)	1.27 (0.29)
*Overall score across above domains*	*1.32 (0.25)*	*1.37 (0.20)*	*1.26 (0.31)*
**HIT System Functionality Overall Score ^f^, mean (sd) with implementation = 0 for absent, 1 for partial, or 2 for full**	1.36 (0.67)	1.11 (0.57)	1.69 (0.70)

Notes: ^a^ Values reported are percentages. Percentages may not sum to 100 due to rounding. ^b^ In this study, we included as primary care providers (PCPs) all individuals serving in clinical role categories defined in the Harvard AIC initiative, including attending physicians (physicians, nurse practitioners, and physician assistants), resident physicians, and other patient-facing clinical personnel (including specialist physicians, mental, and behavioral health providers, medical assistants, registered and other nonregistered nurses, and allied health professionals). ^c^ Self-described based on survey results but not collected among resident physicians. ^d^ Headcount number of MDs. ^e^ Operational care process functionality rescaled from a four-point response scale (1 = Not implemented, 2 = Considering, 3 = Partially implemented, 4 = Fully implemented). ^f^ HIT: Health Information Technology (HIT) Functionality Score was based on 42 items pertaining to the degree to which HIT was being used for clinical decision support, lab ordering, imaging and procedure management, meaningful use stage 2 requirements, referrals, patient portal, recording aspects of patient complexity, and patient registries. ^†^ ED: emergency department.

**Table 2:** Descriptive statistics for Team Dynamics and for Operational Care Process and Health Information Technology (HIT) Functionalities by Primary Care Practice Site ^a^.

Site ID	Operational Care Process Functionality																HIT ^c^ Functionality (Mean, SD)		OUTCOME Team Dynamics (Mean, SD)	
	Domain 1		Domain 2		Domain 3		Domain 4		Domain 5		Domain 6		Domain 7		Domain 8					
	Appointment and Referral Mgmt ^b^ and Tracking				Abnormal Test Result Management (Mean, SD)		Cancer Screening and Medication Mgmt				Patient-Centered Care (Mean, SD)		Patient Safety (Mean, SD)		Care Transitions to EDs * or Hospitals (Mean, SD)					
	For High-Risk Patients (Mean, SD ^†^)		For Routine Patients (Mean, SD)				For High-Risk Patients (Mean, SD)		For Routine Patients (Mean, SD)											
1	2.00	0	1.50	0.84	1.50	1.00	2.00	0	2.00	0	1.40	0.89	1.29	0.49	1.75	0.71	0.79	0.34	3.57	0.60
2	1.00	0	0.33	0.52	1.75	0.50	1.00	0	1.00	0	1.80	0.45	1.29	0.76	1.25	0.89	0.79	0.35	3.61	0.55
3	1.00	1.00	0.50	0.84	1.50	0.58	1.00	0	0	0	1.60	0.55	1.57	0.79	1.00	1.07	1.32	1.24	3.80	0.52
4	1.00	1.00	0.50	0.55	1.00	1.15	0.00	0	0	0	1.20	0.45	1.71	0.76	1.13	0.83	1.20	1.06	3.81	0.50
5	1.33	0.58	0.83	0.41	1.50	0.58	0.50	0.58	1.50	0.71	1.60	0.55	1.71	0.76	1.75	0.46	0.61	0.42	3.81	0.52
6	2.00	0.00	1.00	0.89	1.50	0.58	1.00	0	1.00	0	1.00	0	1.29	0.76	0.88	0.35	0.51	0.61	3.85	0.46
7	1.00	1.00	0.50	0.84	2.00	0	0.75	0.50	1.00	0	2.00	0	1.57	0.79	1.38	0.74	0.98	1.22	3.34	0.66
8	1.33	1.15	1.00	0.89	1.00	0.82	0.75	0.96	1.00	0	1.60	0.55	1.71	0.76	1.25	0.89	1.03	0.63	3.70	0.65
9	0.67	0.58	0.33	0.52	1.25	0.50	0.75	0.96	1.50	0.71	0.80	0.45	1.14	0.69	1.13	0.64	0.60	0.34	4.26	0.61
10	2.00	0	1.17	0.98	0.75	0.50	0.25	0.50	1.00	0	1.40	0.55	1.14	0.69	0.88	0.83	1.96	2.91	3.73	0.65
11	0.67	0.58	0.67	0.52	1.75	0.50	1.25	0.96	1.50	0.71	1.60	0.55	1.83	0.79	1.63	0.52	2.15	2.89	3.92	0.49
12	1.00	0	0.83	0.41	2.00	0	1.75	0.50	1.00	0	1.20	0.45	1.71	0.76	1.88	0.35	1.05	0.63	3.62	0.65
13	1.00	0	0.83	0.41	1.75	0.50	1.00	0	1.00	0	0.80	0.45	1.71	0.76	1.13	0.64	1.97	1.62	3.50	0.72
14	1.67	0.58	0.50	0.84	2.00	0	2.00	0	2.00	0	0.60	0.89	1.71	0.76	1.25	1.04	2.53	3.13	3.96	0.58
15	0.33	0.58	1.17	0.98	2.00	0	2.00	0	2.00	0	1.80	0.45	0.86	0.90	1.75	0.46	2.18	2.93	3.51	0.55
16	2.00	0.00	1.83	0.41	2.00	0	2.00	0	2.00	0	1.60	0.55	2.00	0.76	1.88	0.35	2.22	2.89	3.76	0.50
17	1.33	0.58	1.00	0.63	2.00	0	1.00	0	1.00	0	1.80	0.45	0.71	0.49	1.38	0.52	1.22	1.56	4.14	0.77
18	2.00	0.00	1.80	0.45	2.00	0.00	2.00	0.00	2.00	0.00	0.80	0.45	1.86	0.38	1.75	0.46	0.61	0.45	3.45	0.75
19	0.67	0.58	1.00	1.10	1.75	0.50	1.50	0.58	2.00	0.00	1.00	1.00	1.83	0.79	1.38	0.74	2.10	2.99	3.83	0.58
Overall Mean	1.26	0.54	0.91	0.45	1.63	0.39	1.18	0.63	1.44	0.46	1.34	0.42	1.47	0.32	1.38	0.33	1.36	0.67	3.69	0.63

Notes: ^a^ Operational care process functionality rescaled from a four-point response scale (1 = *Not implemented, 2 = Considering, 3 = Partially implemented, 4 = Fully implemented*) into a three-point scale with implementation = *0 for absent, 1 for partial, 2 for full*. Items regarding HIT (health information technology) functionality used a three-point response scale (*0 = Not implemented, 1 = Partially implemented, 2 = Fully implemented*); items related to team dynamics used a five-point Likert response scale (*1 = Strongly disagree* to *5 = Strongly agree*). ^b^ mgmt: management. ^c^ HIT: health information technology; HIT functionality included 42 items pertaining to eight domains, including clinical decision support, lab ordering, image screening and management, meaningful use stage 2 requirements, referrals, patient portal, recording aspects of complexity, and patient registries. ^†^ SD: standard deviation; * ED: emergency department.

**Table 3:** System operational care process and health information technology (HIT) functionalities (conditions) and qualitative comparative analysis scoring (calibration) used to identify key recipes (combinations of factors) for strong team dynamics in primary care practices.

Conditions (Factors)	Description of Conditions	Calibration Results—Anchor Points/Thresholds Identified ^†^ Upper (Fully or Nearly Fully *Inside* the Target Set); Crossover (Point of Maximum Ambiguity); Lower (Fully or Nearly Fully *Outside* the Target Set)
**Operational Care Process Functionality**		
(1) Appointment and referral management and tracking for high-risk/high-priority patients	The degree to which the practice site has formal systems or processes to track patient arrivals for appointments, receipt of consultation notes by physicians, and referrals for high-risk/high-priority patients.	1.8, 0.6, 0
(2) Appointment and referral management and tracking for routine patients	The degree to which the practice site has formal systems or processes to track patient arrivals for appointments, receipt of consultation notes by physicians, and referrals for routine patients.	2, 1.6, 0.7
(3) Abnormal test results management	The degree to which the practice site has formal systems or processes to ensure that, when test results come back as abnormal, a clinician reviews the abnormal results, notifies the patient, ensures that the patient discusses the result with a physician, and ensures appropriate follow-up.	2, 1.2, 0
(4) Cancer screening and medication management for high-risk/high-priority patients	The degree to which the practice site has formal systems or processes to manage cancer screening tests (e.g., mammograms or colonoscopies) for *high-priority* patients, ensure tests are completed, ensure follow-up, and adjust medications as needed.	2, 1.2, 0
(5) Cancer screening and medication management for routine patients	The degree to which the practice site has formal systems or processes to manage cancer screening tests (e.g., mammograms or colonoscopies) for *routine* patients, ensure tests are completed, ensure follow-up, and adjust medications as needed.	2, 1.3, 0
(6) Patient-centered care	The degree to which the practice site has formal systems or processes to solicit, receive, and act on patient feedback, as well as conduct patient evaluations relating to their experience or satisfaction.	2, 1.5, 0.7
(7) Patient safety	The degree to which the practice site has formal systems or processes to ensure reporting of patient-safety events, near misses, or concerns; analyze data from the reporting to increase understanding; aggregate and track patient safety events over time to identify patterns; incorporate lessons from patient safety reports into plans to improve patient safety; and facilitate learning-oriented discussions about patient-safety events.	1.8, 1.3, 0.8
(8) Care transitions to EDs or hospitals	The degree to which the practice site has formal systems or processes to ensure patient information is transited to and received from admitting hospitals, follow patients during hospitalizations, coordinate discharge plans, and schedule necessary follow-up appointments.	1.9, 1.3, 0.7
HIT * Functionality (HIT)	The degree to which the practice site has a health information-technology system to facilitate lab ordering, image screening and management, referrals, and recording of elements of patient complexity, as well as a patient portal and patient registries to support patient engagement.	3.9, 2.9, 2.0
**OUTCOME: Team Dynamics**	The degree to which care providers perceive themselves as delivering patient care in a “team”.	4.2, 3.8, 3.4

Notes: * HIT: health information technology; ^†^ In the calibration, the raw data were rescaled into set membership scores ranging from 0 to 1 for each condition (factor) to indicate the extent to which this condition was met for each practice site with 0 = condition fully unmet and 1 = condition fully met. As required by QCA, three values (anchor points) of the variable were identified: (1) upper anchor point (the threshold representing fully or nearly fully inside the target set), (2) the crossover point, which is the point of “maximum ambiguity” determining whether a case is inside or outside of the target set, and (3) lower anchor point (the threshold representing fully or nearly fully outside the target set).

**Table 4:** Calibrated data table for the Qualitative Comparative Analysis ^†^.

Site ID	Operational Care Process Functionality								HIT ^b^ Functionality	OUTCOME: Team Dynamics
	Domain 1	Domain 2	Domain 3	Domain 4	Domain 5	Domain 6	Domain 7	Domain 8		
	Appointment and Referral Mgmt ^a^ and Tracking		Abnormal Test Results Mgmt	Cancer Screening and Medication Management		Patient-Centered Care	Patient Safety	Care Transitions to EDs * or Hospitals		
	For High-Risk Patients	For Routine Patients		For High-Risk Patients	For Routine Patients					
1	0.95	0.05	0.45	0.95	0.95	0.68	0.31	0.94	0.93	0.99
2	0.43	0.38	0.75	0.62	0.62	0.90	0.31	0.56	0.96	0.47
3	0.11	0.15	0.45	0.62	0.62	0.82	0.61	0.18	0.16	0.49
4	0.23	0.15	0.25	0.05	0.05	0.50	0.78	0.36	0.14	0.40
5	0.69	0.64	0.45	0.15	0.79	0.82	0.78	0.94	0.69	0.84
6	0.69	0.72	0.45	0.62	0.62	0.27	0.31	0.08	0.07	0.43
7	0.95	0.55	0.95	0.62	0.38	0.95	0.61	0.71	0.55	0.45
8	0.69	0.72	0.25	0.62	0.38	0.82	0.78	0.56	0.45	0.42
9	0.11	0.15	0.34	0.62	0.62	0.12	0.21	0.36	0.69	0.84
10	0.69	0.55	0.17	0.15	0.38	0.68	0.21	0.08	0.71	0.63
11	0.95	0.84	0.75	0.90	0.62	0.82	0.88	0.89	0.68	0.49
12	0.05	0.05	0.95	0.90	0.79	0.50	0.78	0.97	0.95	0.41
13	0.43	0.64	0.75	0.62	0.62	0.12	0.78	0.36	0.88	0.87
14	0.23	0.38	0.95	0.95	0.95	0.05	0.78	0.56	0.59	0.92
15	0.11	0.89	0.95	0.95	0.95	0.90	0.08	0.94	0.85	0.45
16	0.95	0.96	0.95	0.95	0.95	0.82	0.95	0.97	0.52	0.96
17	0.69	0.78	0.95	0.62	0.62	0.90	0.05	0.71	0.64	0.96
18	0.95	0.50	0.95	0.95	0.95	0.12	0.89	0.94	0.29	0.38
19	0.23	0.78	0.75	0.79	0.95	0.27	0.88	0.71	0.73	0.62

Notes: ^†^ Researchers used the statistical properties of the data to rescale (“calibrate”, in the terminology of QCA) the results onto a 0.0 to 1.0 scale for each observed case to indicate the extent to which these likely explanatory factors (“conditions”, in the terminology of QCA) are present for each practice with 1 = condition is fully met, and 0 = condition is fully unmet. This table summarizes the calibrated scores for all the conditions and the outcome for each primary care practice site; ^a^ mgmt: management; ^b^ HIT: health information technology. * ED: emergency department.

**Table 5:** Qualitative Comparative Analysis results (n = 19 Academic Innovations Collaborative primary care practices).

Recipes, i.e., Combinations of Factors (Conditions) Leading to Strong Team Dynamics among Primary Care Providers	Consistency ^†^	Raw Coverage ^††^	Unique Coverage ^£^	Observations with Strong Membership in This Recipe ^§^
Recipe 1: Abnormal test mgmt * Cancer screening/medication mgmt ^a^ for high-priority patients * Patient-centered care * Care transitions * HIT	0.94	0.37	0.10	2, 15, 17
Recipe 2: Abnormal test mgmt * Cancer screening/medication mgmt for high-priority patients * Patient safety processes * Care transitions * HIT	0.97	0.38	0.11	14, 19
Total Solution	0.95	0.48	NA	NA

Notes: ^a^ mgmt: management. ^†^ Consistency: indicates the strengths of the relationship. A consistency score of 1.0 for sufficient conditions indicates that the outcome occurs whenever the condition presents. A consistency score of 0.9 for sufficient conditions indicates that a condition or a combination of conditions is “almost always sufficient” for outcome occurrence. ^††^ Coverage: measures the empirical relevancy of the identified relationship (i.e., the fraction of cases in the empirical data to which the relationship applies). Coverage is measured in two ways: raw coverage and unique coverage. Raw coverage identifies the cases described by a given combination of conditions (“recipes”). ^£^ Unique coverage identifies those cases that exclusively belong to a single recipe. A recipe with high raw coverage indicates that this combination of conditions can specify many cases in which the relationship applies, while a recipe with high unique coverage specifies many cases that are not also explained by other recipes. * “AND.” **^§^** Observations with strong membership in this recipe: primary care practices which exhibited the reported combinations of factors (conditions) for the occurrence of outcome (great team dynamics among primary care providers in practices).

**Figure A1:**
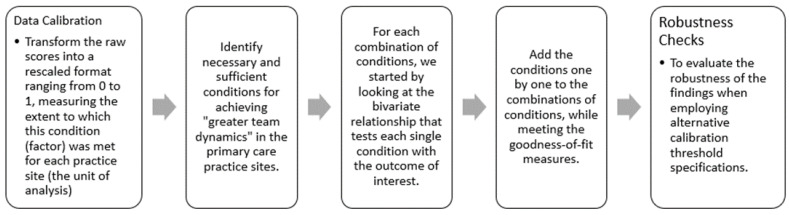
Visualized QCA steps.

## Data Availability

Raw data were generated at the Harvard AIC–CARES Initiative. Derived data supporting the findings of this study are available from the corresponding author LL on request.

## Appendix A

**Table A1:** Team Dynamics (33 items): Please indicate your agreement or disagreement with the following statements about your “team” ^†^.

Factor Name	Item Number and Text
Conditions for team effectiveness	1. Membership on my team changes so frequently that we don’t really have a team.
	2. My team has the right “mix” of members—a group of people who bring different clinical perspectives and experiences to the work.
	3. It is clear what is—and what is not—acceptable behavior on my team.
	4. Our practice recognizes and reinforces teams that perform well.
Shared understanding	5. My team has goals that are clear, useful and appropriate to my practice.
	6. There is a real desire among team members to work collaboratively.
	7. If asked, I could explain every team member’s role and how they overlap.
	8. My team encourages patients to be active participants in decisions about their care.
	9. My team does a good job of helping patients understand their care plan.
	10. The patient’s needs and preferences are treated as an essential part of my team’s decisions.
Processes for accountability	11. Each team member shares accountability for team decisions and outcomes.
Processes for communication and information exchange	12. My team has developed effective strategies for sharing patient treatment goals among team members.
	13. Relevant information about changes in patient status or care plan is reported to the appropriate team member in a timely manner.
	14. All team members effectively use the patient health record as a communication tool.
	15. My team addresses patients’ concerns effectively through team meeting and discussion.
	16. Team meetings provide an open, comfortable, safe place to discuss concerns.
Processes for conflict resolution	17. When team members disagree, all points of view are considered before deciding on a solution.
	18. My team has an effective process for conflict management.
Acting and feeling like a team	19. Members of my team depend on each other for their special knowledge and expertise.
	20. Overall, members of our team do a very good job of coordinating their different patient-related jobs and activities.
	21. I regularly communicate with other members of my team.
	22. Members of my team act upon the information I communicate to them.
	23. Members of my team show respect for each other’s roles and expertise.
	24. Members of my team really trust each other’s work and contributions related to patient care.
Perceived team effectiveness	25. The way my team members interact makes the delivery of care highly efficient.
	26. The way my team members interact is very good for the quality of patient care.
	27. Working on a team like mine keeps members of my team enthusiastic and interested in their jobs.
	28. I feel integral to my team.
	29. I experience excellent teamwork with the members of my team.
Learning processes ^††^	30. My team discusses its weaknesses in order to do better work in the future.
	31. My team is always searching for new ways to address problems.
	32. Members of my team support new ideas by helping to test them in practice.
	33. My team tracks its performance in order to do its best work.

Notes: ^†^ Respondents were asked in the survey’s opening question to define their primary care team by selecting the types of people with whom they frequently collaborate when providing patient care at their practice. They were then asked to consider this group of people as their “team” in answering subsequent questions. ^††^ The original survey contains the seven factors [12], and the modified survey in this study included a new factor on learning processes. Prior study by the AIC–CARES evaluation team confirmed that the modification did not adversely impact the internal consistency (i.e., Cronbach’s alphas) or discriminant validity (Pearson correlations between factors) [14].
